# Tris{2-meth­oxy-6-[(4-methyl­phen­yl)iminiometh­yl]phenolate-κ^2^ *O*,*O*′}tris­(thio­cyanato-κ*N*)lanthanum(III)

**DOI:** 10.1107/S1600536809049113

**Published:** 2009-11-21

**Authors:** Jian-Feng Liu, Jin-Bei Shen, Jia-Lu Liu, Guo-Liang Zhao

**Affiliations:** a Zhejiang Key Laboratory for Reactive Chemistry on Solid Surfaces, Institute of Physical Chemistry, Zhejiang Normal University, Jinhua, Zhejiang 321004, People’s Republic of China, and College of Chemistry and Life Science, Zhejiang Normal University, Jinhua 321004, Zhejiang, People’s Republic of China

## Abstract

In the title compound, [La(NCS)_3_(C_15_H_15_NO_2_)_3_], the metal centre is nine-coordinated by six O atoms from three zwitterionic Schiff base 2-meth­oxy-6-[(4-methyl­phen­yl)iminio­meth­yl]phenolate (*L*) ligands and three terminal N atoms of the thio­cyanate ions in a monocapped square-anti­prismatic environment. The *L* ligands chelate the La^III^ ion with strong La—O(deprotonated phenolic) bonds [2.435 (3)–2.464 (3) Å] and significantly longer La—O(meth­oxy) bonds [2.801 (3)–2.831 (3) Å]. The La—N bond lengths range from 2.541 (4) to 2.605 (4) Å.

## Related literature

For synthetic background, see: Yeap *et al.* (2003 ▶). For the structures of related Ce^III^ and Tb(III) complexes, see: Liu *et al.* (2009 ▶); Zhao *et al.* (2007 ▶).
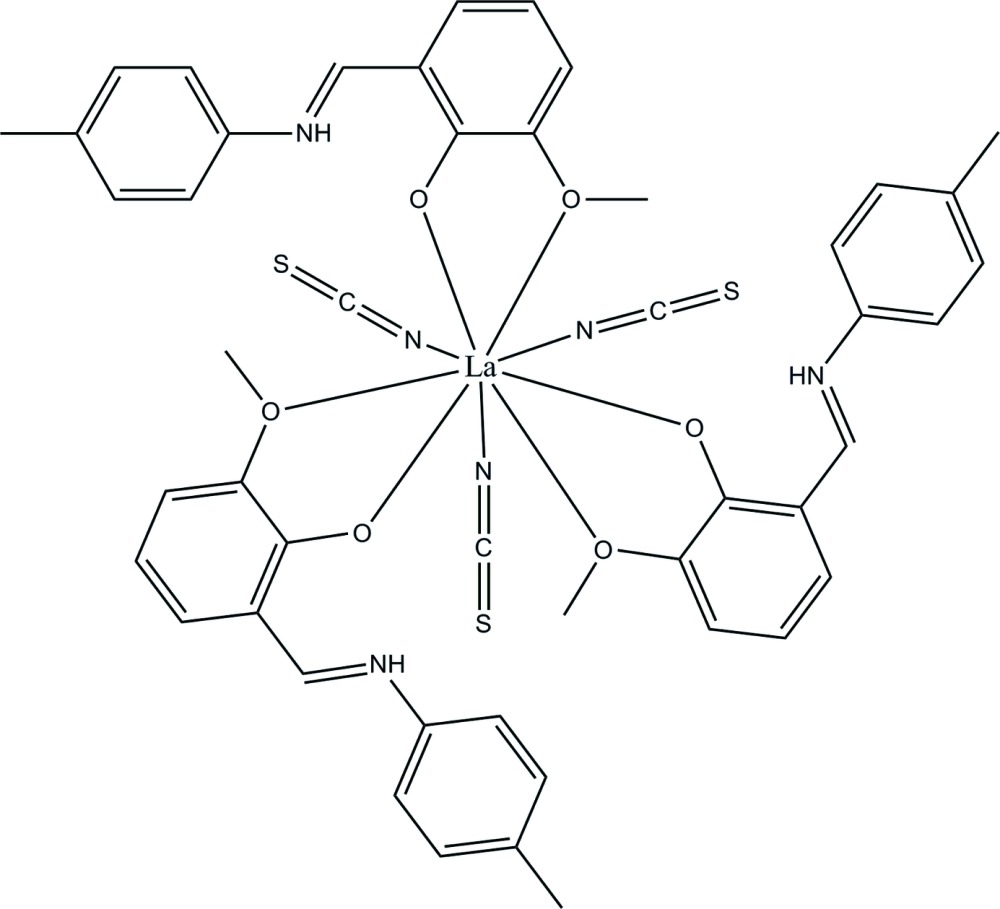


## Experimental

### 

#### Crystal data

[La(NCS)_3_(C_15_H_15_NO_2_)_3_]
*M* *_r_* = 1036.99
Monoclinic, 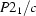

*a* = 16.7056 (7) Å
*b* = 14.2407 (6) Å
*c* = 22.2167 (10) Å
β = 106.156 (2)°
*V* = 5076.6 (4) Å^3^
*Z* = 4
Mo *K*α radiation
μ = 1.02 mm^−1^
*T* = 296 K
0.17 × 0.09 × 0.06 mm

#### Data collection

Bruker APEXII CCD area-detector diffractometer
Absorption correction: multi-scan (*SADABS*; Sheldrick, 1996 ▶) *T*_min_ = 0.899, *T*_max_ = 0.942
67027 measured reflections
8926 independent reflections
6157 reflections with *I* > 2σ(*I*)
*R*_int_ = 0.079

#### Refinement

*R*[*F* ^2^ > 2σ(*F* ^2^)] = 0.043
*wR*(*F* ^2^) = 0.117
*S* = 1.03
8926 reflections
577 parameters
12 restraints
H-atom parameters constrained
Δρ_max_ = 0.53 e Å^−3^
Δρ_min_ = −0.54 e Å^−3^

### 

Data collection: *APEX2* (Bruker, 2006 ▶); cell refinement: *SAINT* (Bruker, 2006 ▶); data reduction: *SAINT*; program(s) used to solve structure: *SHELXS97* (Sheldrick, 2008 ▶); program(s) used to refine structure: *SHELXL97* (Sheldrick, 2008 ▶); molecular graphics: *SHELXTL* (Sheldrick, 2008 ▶); software used to prepare material for publication: *SHELXL97*.

## Supplementary Material

Crystal structure: contains datablocks I, global. DOI: 10.1107/S1600536809049113/pv2228sup1.cif

Structure factors: contains datablocks I. DOI: 10.1107/S1600536809049113/pv2228Isup2.hkl

Additional supplementary materials:  crystallographic information; 3D view; checkCIF report

## Figures and Tables

**Table 1:** Hydrogen-bond geometry (Å, °)

*D*—H⋯*A*	*D*—H	H⋯*A*	*D*⋯*A*	*D*—H⋯*A*
N1—H1*D*⋯O2	0.86	1.86	2.559 (4)	138
N2—H2*A*⋯O4	0.86	1.90	2.588 (4)	136
N3—H3*B*⋯O6	0.86	1.87	2.570 (4)	138

**Fig. 1.**
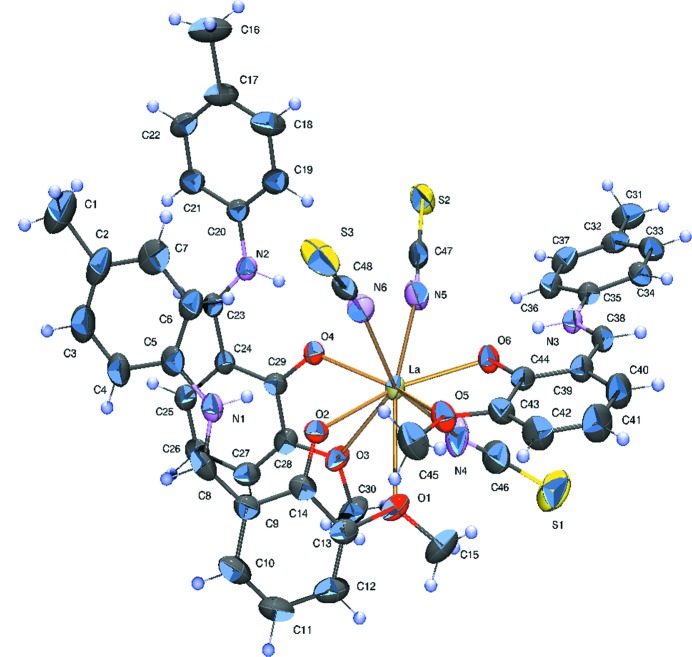
The molecular structure of the title complex, showing the atom-labeling scheme. Displacement ellipsoids are drawn at the 30% probability level.

**Fig. 2.**
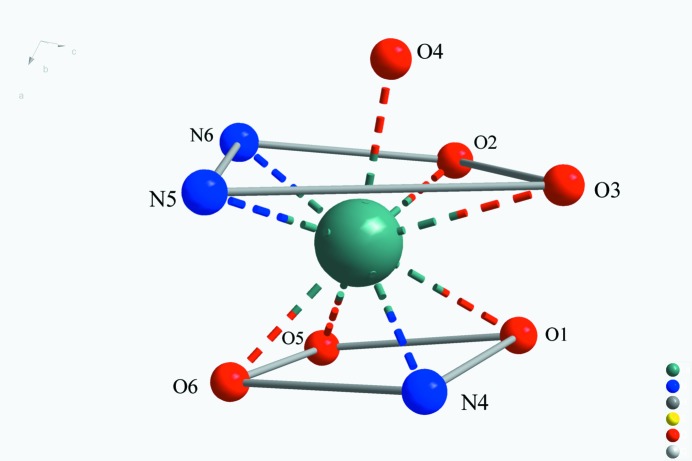
The coordination environment of the lanthanum(III) atom, showing the monocapped square antiprism.

## supplementary crystallographic information

### Comment

The rare earth complexes prepared by ligands derived from *o*-vanillin have been reported in the past decades due to the intriguing biological activities of *o*-vanillin and the varied structures and behaviors of the Schiff bases (Yeap *et al.*, 2003). For these reasons, we have been engaged in the syntheses of new analogous Schiff bases derived from *o*-vanillin and their rare metal complexes. For the past few years we have synthesized and reported several Schiff complexes (Zhao *et al.*, 2007; Liu *et al.*, 2009). Herein, we describe the crystal structure of a new lanthanum(III) complex, (I).

The structure of the title compound is shown in Fig. 1.The compound (I) is composed of three 2-[(4-methylphenyl-imino)methyl]-6-methoxyphenol (H*L*) ligands and three independent thiocyanate ions. The La^III^ is nine-coordinated by three N atoms from three thiocyanate ions and six O atoms from the H*L* ligands. The three H*L* ligands chelate the La^III^ ion with methoxy O atoms and deprotonated phenolic O atoms. The shorter La—O bonds involve the phenolic oxygen atoms (2.435 (3)–2.464 (3) Å). The La—O (methoxy) links are significantly longer (2.801 (3)–2.831 (3) Å) than the La—O (phenolic) distances. The La—N bonds vary from 2.541 (4)Å to 2.605 (4) Å. These bond lengths correspond to those previously observed (Liu *et al.*, 2009). While in contract to the isomorphous Ce^III^ complex, the La—*X* (*X* = O/N) are slightly longer than the corresponding distances reported in the Ce^III^ complex (Liu *et al.*, 2009), which can be attribute to a decrease in the ionic radii from La^III^ to Ce^III^ due to the lanthanide contraction. Because of the geometric and chemical environment requirements of the chelating groups the coordination geometry deviates considerably from the distorted capped square antiprism geometry (Fig. 2). In the H*L* ligands, the protons of the phenolic groups are considered to have transferred to *N*-imine atoms, which are involved in intramolecular hydrogen bonds (Table 1) that probably forces them to assume nearly planar conformations.

### Experimental

*p*-Toluidine (1.07 g, 10 mmol) was added to *o*-vanillin (1.52 g, 10 mmol) in methanol (20 ml) to give an orange solution. A solid product (H*L*) was separated from the solution after stirring for about 10 min. and was purified by recrystallization from ethanol.

To a methanol solution of *N*-3-methoxysalicylidene-*p*-toluidine (H*L*) (3 mmol, 10 ml) was dropped 1 mmol La(Cl_3_)_3_.6H_2_O (dissolved in methanol) under stirring condition and the mixture solution was still stirred at room temperature for 8 h to give a purplish red solution. The deposit was filtered out and the solution was kept for evaporating. The red crystals of the title compound (I) were formed after several days.

### Refinement

The H atoms bonded to C and N atoms were positioned geometrically and refined using a riding model with C—H distances: 0.96, 0.93 and 0.86 Å for aliphatic, aromatic and imino H-atoms, respectively, and *U*_iso_(H) = 1.5*U*_eq_(C-aliphatic) or 1.2*U*_eq_(the rest of the parent atoms).

### Crystal data

**Table:**

[La(NCS)_3_(C_15_H_15_NO_2_)_3_]	*F*(000) = 2112
*M**_r_* = 1036.99	*D*_x_ = 1.357 Mg m^−^^3^
Monoclinic, *P*2_1_/*c*	Mo *K*α radiation, λ = 0.71073 Å
Hall symbol: -P 2ybc	Cell parameters from 6660 reflections
*a* = 16.7056 (7) Å	θ = 1.3–25.0°
*b* = 14.2407 (6) Å	µ = 1.02 mm^−^^1^
*c* = 22.2167 (10) Å	*T* = 296 K
β = 106.156 (2)°	Block, red
*V* = 5076.6 (4) Å^3^	0.17 × 0.09 × 0.06 mm
*Z* = 4	

### Data collection

**Table:**

Bruker APEXII CCD area-detector diffractometer	8926 independent reflections
Radiation source: fine-focus sealed tube	6157 reflections with *I* > 2σ(*I*)
graphite	*R*_int_ = 0.079
phi and ω scans	θ_max_ = 25.0°, θ_min_ = 1.7°
Absorption correction: multi-scan (*SADABS*; Sheldrick, 1996)	*h* = −19→19
*T*_min_ = 0.899, *T*_max_ = 0.942	*k* = −16→16
67027 measured reflections	*l* = −26→26

### Refinement

**Table:**

Refinement on *F*^2^	Primary atom site location: structure-invariant direct methods
Least-squares matrix: full	Secondary atom site location: difference Fourier map
*R*[*F*^2^ > 2σ(*F*^2^)] = 0.043	Hydrogen site location: inferred from neighbouring sites
*wR*(*F*^2^) = 0.117	H-atom parameters constrained
*S* = 1.03	*w* = 1/[σ^2^(*F*_o_^2^) + (0.0608*P*)^2^] where *P* = (*F*_o_^2^ + 2*F*_c_^2^)/3
8926 reflections	(Δ/σ)_max_ = 0.002
577 parameters	Δρ_max_ = 0.53 e Å^−^^3^
12 restraints	Δρ_min_ = −0.54 e Å^−^^3^

### Special details

**Table:**

Experimental. H*L* product: yield = 80%, (m.p. 373-374 K). Analysis calculated for C_15_H_15_NO_2_: C 74.66, H 6.27, N 5.81%; found: C 74.62, H 6.31, N 5.77%.
Geometry. All e.s.d.s (except the e.s.d. in the dihedral angle between two l.s. planes) are estimated using the full covariance matrix. The cell e.s.d.s are taken into account individually in the estimation of e.s.d.s in distances, angles and torsion angles; correlations between e.s.d.s in cell parameters are only used when they are defined by crystal symmetry. An approximate (isotropic) treatment of cell e.s.d.s is used for estimating e.s.d.s involving l.s. planes.
Refinement. Refinement of *F*^2^ against ALL reflections. The weighted *R*- factor *wR* and goodness of fit *S* are based on *F*^2^, conventional *R*-factors *R* are based on *F*, with *F* set to zero for negative *F*^2^. The threshold expression of *F*^2^ > σ(*F*^2^) is used only for calculating *R*- factors(gt) *etc*. and is not relevant to the choice of reflections for refinement. *R*-factors based on *F*^2^ are statistically about twice as large as those based on *F*, and *R*-factors based on ALL data will be even larger.

### Fractional atomic coordinates and isotropic or equivalent isotropic displacement parameters (Å^2^)

**Table:**

	*x*	*y*	*z*	*U*_iso_*/*U*_eq_	
La	−0.210769 (14)	0.726484 (17)	0.609038 (11)	0.04975 (11)	
N4	−0.1095 (3)	0.6439 (4)	0.7005 (2)	0.1172 (19)	
N5	−0.2191 (3)	0.5653 (3)	0.5522 (2)	0.0833 (13)	
N6	−0.2775 (3)	0.7778 (3)	0.49480 (19)	0.0763 (12)	
N1	−0.4571 (2)	0.8997 (3)	0.56913 (18)	0.0626 (10)	
H1D	−0.4117	0.8763	0.5646	0.075*	
N2	−0.4810 (2)	0.6228 (2)	0.49819 (16)	0.0525 (9)	
H2A	−0.4277	0.6261	0.5129	0.063*	
N3	0.0256 (2)	0.5771 (2)	0.58132 (15)	0.0506 (8)	
H3B	−0.0231	0.5950	0.5826	0.061*	
S1	0.05539 (13)	0.64120 (17)	0.77073 (12)	0.1670 (10)	
S2	−0.25313 (9)	0.39641 (11)	0.48846 (8)	0.1041 (6)	
S3	−0.33355 (13)	0.89946 (14)	0.39497 (7)	0.1237 (7)	
O1	−0.1648 (2)	0.8658 (2)	0.70273 (14)	0.0755 (9)	
O2	−0.30215 (17)	0.8609 (2)	0.61258 (13)	0.0596 (8)	
O3	−0.28240 (19)	0.7013 (2)	0.70787 (13)	0.0676 (9)	
O4	−0.35101 (16)	0.6605 (2)	0.59004 (13)	0.0574 (7)	
O5	−0.1183 (2)	0.8823 (2)	0.58584 (15)	0.0702 (9)	
O6	−0.08077 (17)	0.70762 (18)	0.58104 (15)	0.0592 (8)	
C1	−0.7431 (4)	0.8012 (5)	0.3705 (3)	0.138 (3)	
H1A	−0.7935	0.8086	0.3827	0.207*	
H1B	−0.7374	0.7368	0.3596	0.207*	
H1C	−0.7452	0.8405	0.3349	0.207*	
C2	−0.6683 (4)	0.8295 (4)	0.4251 (3)	0.0940 (19)	
C3	−0.6762 (4)	0.8608 (4)	0.4822 (4)	0.102 (2)	
H3A	−0.7288	0.8654	0.4885	0.122*	
C4	−0.6082 (3)	0.8849 (4)	0.5290 (3)	0.0880 (17)	
H4A	−0.6147	0.9059	0.5671	0.106*	
C5	−0.5298 (3)	0.8787 (3)	0.5211 (2)	0.0658 (13)	
C6	−0.5196 (3)	0.8483 (3)	0.4648 (3)	0.0721 (14)	
H6A	−0.4667	0.8443	0.4590	0.086*	
C7	−0.5893 (4)	0.8237 (4)	0.4171 (3)	0.0860 (16)	
H7A	−0.5828	0.8029	0.3791	0.103*	
C8	−0.4494 (3)	0.9497 (3)	0.6194 (3)	0.0732 (14)	
H8A	−0.4963	0.9801	0.6246	0.088*	
C9	−0.3738 (3)	0.9599 (3)	0.6662 (2)	0.0667 (12)	
C10	−0.3694 (4)	1.0160 (4)	0.7207 (3)	0.0988 (19)	
H10A	−0.4163	1.0479	0.7245	0.119*	
C11	−0.2973 (5)	1.0221 (5)	0.7660 (3)	0.115 (2)	
H11A	−0.2946	1.0592	0.8010	0.138*	
C12	−0.2264 (4)	0.9742 (4)	0.7619 (3)	0.0988 (19)	
H12A	−0.1771	0.9794	0.7940	0.119*	
C13	−0.2288 (3)	0.9197 (4)	0.7112 (2)	0.0681 (13)	
C14	−0.3024 (3)	0.9116 (3)	0.6611 (2)	0.0600 (12)	
C15	−0.0839 (3)	0.8786 (4)	0.7454 (3)	0.105 (2)	
H15A	−0.0850	0.9312	0.7722	0.157*	
H15B	−0.0442	0.8900	0.7223	0.157*	
H15C	−0.0683	0.8230	0.7704	0.157*	
C16	−0.6000 (4)	0.5597 (5)	0.2350 (2)	0.121 (2)	
H16A	−0.6585	0.5458	0.2244	0.182*	
H16B	−0.5710	0.5080	0.2228	0.182*	
H16C	−0.5912	0.6154	0.2133	0.182*	
C17	−0.5671 (4)	0.5750 (4)	0.3052 (2)	0.0792 (15)	
C18	−0.4851 (4)	0.5984 (4)	0.3338 (2)	0.0891 (17)	
H18A	−0.4478	0.6046	0.3098	0.107*	
C19	−0.4582 (3)	0.6127 (3)	0.3972 (2)	0.0722 (14)	
H19A	−0.4029	0.6289	0.4157	0.087*	
C20	−0.5110 (3)	0.6035 (3)	0.4338 (2)	0.0535 (11)	
C21	−0.5928 (3)	0.5788 (3)	0.4061 (2)	0.0672 (13)	
H21A	−0.6295	0.5716	0.4305	0.081*	
C22	−0.6200 (3)	0.5650 (3)	0.3422 (2)	0.0722 (14)	
H22A	−0.6752	0.5485	0.3238	0.087*	
C23	−0.5232 (3)	0.6363 (3)	0.5384 (2)	0.0535 (11)	
H23A	−0.5808	0.6302	0.5243	0.064*	
C24	−0.4877 (2)	0.6596 (3)	0.60205 (19)	0.0496 (10)	
C25	−0.5396 (3)	0.6721 (3)	0.6419 (2)	0.0657 (12)	
H25A	−0.5970	0.6647	0.6265	0.079*	
C26	−0.5051 (3)	0.6950 (4)	0.7027 (2)	0.0723 (14)	
H26A	−0.5396	0.7042	0.7286	0.087*	
C27	−0.4193 (3)	0.7053 (3)	0.7275 (2)	0.0668 (13)	
H27A	−0.3968	0.7207	0.7695	0.080*	
C28	−0.3683 (3)	0.6925 (3)	0.6894 (2)	0.0567 (11)	
C29	−0.4010 (3)	0.6711 (3)	0.62505 (19)	0.0498 (10)	
C30	−0.2426 (3)	0.7061 (5)	0.7742 (2)	0.097 (2)	
H30A	−0.2830	0.6941	0.7967	0.145*	
H30B	−0.2192	0.7675	0.7847	0.145*	
H30C	−0.1992	0.6598	0.7854	0.145*	
C31	0.0656 (4)	0.1795 (3)	0.5828 (2)	0.0909 (17)	
H31A	0.1178	0.1629	0.5756	0.136*	
H31B	0.0208	0.1522	0.5507	0.136*	
H31C	0.0639	0.1563	0.6230	0.136*	
C32	0.0564 (3)	0.2851 (3)	0.5814 (2)	0.0656 (13)	
C33	0.1161 (3)	0.3429 (3)	0.5718 (2)	0.0671 (13)	
H33A	0.1639	0.3166	0.5652	0.081*	
C34	0.1085 (3)	0.4391 (3)	0.57147 (19)	0.0597 (11)	
H34A	0.1506	0.4770	0.5648	0.072*	
C35	0.0380 (3)	0.4784 (3)	0.58112 (18)	0.0498 (10)	
C36	−0.0249 (3)	0.4219 (3)	0.5902 (2)	0.0641 (12)	
H36A	−0.0730	0.4484	0.5962	0.077*	
C37	−0.0153 (3)	0.3256 (3)	0.5903 (2)	0.0713 (14)	
H37A	−0.0574	0.2872	0.5964	0.086*	
C38	0.0777 (3)	0.6433 (3)	0.5799 (2)	0.0617 (12)	
H38A	0.1307	0.6265	0.5780	0.074*	
C39	0.0589 (3)	0.7394 (3)	0.5810 (2)	0.0590 (12)	
C40	0.1201 (3)	0.8081 (4)	0.5825 (3)	0.0894 (17)	
H40A	0.1733	0.7901	0.5816	0.107*	
C41	0.1016 (4)	0.9003 (4)	0.5854 (3)	0.101 (2)	
H41A	0.1421	0.9454	0.5862	0.121*	
C42	0.0226 (4)	0.9281 (3)	0.5870 (3)	0.0863 (16)	
H42A	0.0112	0.9917	0.5894	0.104*	
C43	−0.0379 (3)	0.8647 (3)	0.5853 (2)	0.0601 (12)	
C44	−0.0220 (3)	0.7679 (3)	0.5825 (2)	0.0525 (11)	
C45	−0.1475 (3)	0.9774 (3)	0.5781 (3)	0.0929 (18)	
H45A	−0.1044	1.0173	0.5714	0.139*	
H45B	−0.1617	0.9974	0.6150	0.139*	
H45C	−0.1957	0.9813	0.5426	0.139*	
C46	−0.0433 (4)	0.6402 (5)	0.7283 (3)	0.1051 (19)	
C47	−0.2342 (3)	0.4964 (4)	0.5250 (2)	0.0677 (13)	
C48	−0.3001 (3)	0.8275 (4)	0.4537 (2)	0.0673 (13)	

### Atomic displacement parameters (Å^2^)

**Table:**

	*U*^11^	*U*^22^	*U*^33^	*U*^12^	*U*^13^	*U*^23^
La	0.04258 (16)	0.05171 (17)	0.05236 (17)	0.00352 (11)	0.00892 (11)	0.00407 (12)
N4	0.069 (3)	0.178 (5)	0.099 (4)	0.047 (3)	0.014 (3)	0.053 (3)
N5	0.067 (3)	0.073 (3)	0.117 (4)	−0.006 (2)	0.039 (3)	−0.022 (3)
N6	0.080 (3)	0.090 (3)	0.058 (3)	0.016 (2)	0.016 (2)	0.012 (2)
N1	0.055 (2)	0.058 (2)	0.075 (3)	0.0132 (18)	0.018 (2)	0.003 (2)
N2	0.046 (2)	0.056 (2)	0.056 (2)	−0.0062 (16)	0.0149 (18)	−0.0005 (17)
N3	0.043 (2)	0.047 (2)	0.062 (2)	0.0027 (17)	0.0141 (17)	0.0006 (17)
S1	0.1077 (15)	0.1510 (19)	0.194 (2)	0.0425 (14)	−0.0378 (15)	−0.0157 (17)
S2	0.0724 (9)	0.0998 (11)	0.1548 (15)	−0.0358 (8)	0.0559 (10)	−0.0605 (11)
S3	0.1769 (18)	0.1342 (15)	0.0745 (10)	0.0800 (14)	0.0587 (11)	0.0406 (10)
O1	0.069 (2)	0.087 (2)	0.057 (2)	−0.0043 (19)	−0.0055 (17)	0.0042 (17)
O2	0.0635 (19)	0.0640 (19)	0.0487 (18)	0.0091 (15)	0.0111 (14)	−0.0055 (15)
O3	0.060 (2)	0.092 (2)	0.0467 (18)	−0.0103 (17)	0.0067 (15)	0.0063 (16)
O4	0.0456 (17)	0.073 (2)	0.0557 (18)	−0.0046 (14)	0.0183 (14)	−0.0054 (15)
O5	0.073 (2)	0.0486 (18)	0.094 (2)	0.0107 (16)	0.0319 (19)	0.0040 (16)
O6	0.0493 (18)	0.0467 (17)	0.086 (2)	−0.0037 (13)	0.0259 (16)	−0.0039 (14)
C1	0.097 (5)	0.106 (5)	0.171 (7)	0.011 (4)	−0.031 (5)	0.004 (5)
C2	0.065 (4)	0.066 (4)	0.125 (5)	0.010 (3)	−0.017 (4)	0.015 (4)
C3	0.069 (4)	0.091 (4)	0.137 (6)	0.021 (3)	0.014 (4)	0.005 (4)
C4	0.064 (4)	0.091 (4)	0.110 (5)	0.023 (3)	0.025 (3)	0.000 (3)
C5	0.066 (3)	0.051 (3)	0.077 (4)	0.019 (2)	0.013 (3)	0.010 (2)
C6	0.064 (3)	0.064 (3)	0.081 (4)	0.012 (2)	0.009 (3)	0.011 (3)
C7	0.095 (5)	0.065 (3)	0.089 (4)	0.015 (3)	0.010 (3)	0.009 (3)
C8	0.084 (4)	0.057 (3)	0.091 (4)	0.014 (3)	0.045 (3)	0.001 (3)
C9	0.075 (3)	0.064 (3)	0.063 (3)	0.003 (3)	0.022 (3)	−0.008 (2)
C10	0.120 (5)	0.091 (4)	0.097 (5)	0.006 (4)	0.049 (4)	−0.028 (4)
C11	0.129 (6)	0.131 (6)	0.086 (5)	−0.005 (5)	0.034 (4)	−0.053 (4)
C12	0.117 (5)	0.113 (5)	0.059 (4)	−0.021 (4)	0.014 (3)	−0.018 (3)
C13	0.077 (4)	0.070 (3)	0.055 (3)	−0.003 (3)	0.015 (3)	0.001 (3)
C14	0.075 (3)	0.055 (3)	0.053 (3)	0.001 (2)	0.022 (3)	0.006 (2)
C15	0.080 (4)	0.111 (5)	0.095 (4)	−0.018 (3)	−0.021 (3)	0.005 (4)
C16	0.171 (7)	0.116 (5)	0.066 (4)	−0.027 (5)	0.018 (4)	−0.026 (4)
C17	0.115 (5)	0.070 (3)	0.051 (3)	−0.016 (3)	0.020 (3)	−0.010 (3)
C18	0.119 (5)	0.095 (4)	0.063 (4)	−0.033 (4)	0.042 (3)	−0.019 (3)
C19	0.068 (3)	0.082 (4)	0.067 (3)	−0.019 (3)	0.020 (3)	−0.014 (3)
C20	0.055 (3)	0.054 (3)	0.050 (3)	−0.004 (2)	0.013 (2)	−0.005 (2)
C21	0.066 (3)	0.070 (3)	0.065 (3)	−0.008 (3)	0.019 (3)	−0.006 (3)
C22	0.069 (3)	0.073 (3)	0.064 (3)	−0.013 (3)	0.001 (3)	−0.013 (3)
C23	0.044 (2)	0.056 (3)	0.063 (3)	−0.003 (2)	0.018 (2)	−0.004 (2)
C24	0.046 (2)	0.050 (2)	0.054 (3)	−0.0017 (19)	0.016 (2)	0.002 (2)
C25	0.052 (3)	0.083 (3)	0.064 (3)	−0.002 (2)	0.020 (2)	−0.002 (3)
C26	0.072 (4)	0.088 (4)	0.067 (3)	−0.004 (3)	0.035 (3)	−0.005 (3)
C27	0.079 (4)	0.070 (3)	0.052 (3)	−0.009 (3)	0.020 (3)	−0.001 (2)
C28	0.058 (3)	0.055 (3)	0.055 (3)	−0.004 (2)	0.014 (2)	0.012 (2)
C29	0.056 (3)	0.048 (2)	0.047 (3)	−0.004 (2)	0.017 (2)	0.0029 (19)
C30	0.079 (4)	0.154 (6)	0.047 (3)	−0.022 (4)	0.000 (3)	0.020 (3)
C31	0.120 (5)	0.053 (3)	0.086 (4)	0.002 (3)	0.007 (3)	−0.006 (3)
C32	0.081 (4)	0.052 (3)	0.056 (3)	0.001 (3)	0.005 (3)	−0.002 (2)
C33	0.077 (3)	0.060 (3)	0.064 (3)	0.015 (3)	0.019 (3)	−0.002 (2)
C34	0.065 (3)	0.059 (3)	0.058 (3)	0.004 (2)	0.022 (2)	0.003 (2)
C35	0.054 (3)	0.046 (2)	0.048 (3)	0.002 (2)	0.013 (2)	0.0016 (19)
C36	0.054 (3)	0.057 (3)	0.077 (3)	0.000 (2)	0.010 (2)	0.001 (2)
C37	0.072 (3)	0.052 (3)	0.081 (4)	−0.014 (3)	0.007 (3)	0.006 (2)
C38	0.051 (3)	0.063 (3)	0.073 (3)	0.001 (2)	0.020 (2)	−0.007 (2)
C39	0.053 (3)	0.047 (3)	0.076 (3)	−0.004 (2)	0.018 (2)	−0.002 (2)
C40	0.068 (4)	0.073 (4)	0.135 (5)	−0.018 (3)	0.041 (3)	−0.011 (3)
C41	0.086 (4)	0.066 (4)	0.159 (6)	−0.029 (3)	0.048 (4)	−0.013 (4)
C42	0.092 (4)	0.047 (3)	0.126 (5)	−0.015 (3)	0.041 (4)	−0.009 (3)
C43	0.069 (3)	0.045 (3)	0.069 (3)	−0.002 (2)	0.025 (2)	−0.002 (2)
C44	0.055 (3)	0.047 (2)	0.055 (3)	−0.004 (2)	0.015 (2)	−0.002 (2)
C45	0.110 (5)	0.052 (3)	0.125 (5)	0.023 (3)	0.046 (4)	0.016 (3)
C46	0.097 (4)	0.124 (5)	0.087 (4)	0.040 (4)	0.014 (4)	0.014 (4)
C47	0.042 (3)	0.071 (3)	0.095 (4)	−0.007 (2)	0.025 (3)	−0.012 (3)
C48	0.076 (3)	0.079 (3)	0.052 (3)	0.016 (3)	0.027 (2)	−0.004 (3)

### Geometric parameters (Å, °)

**Table:**

La—O6	2.435 (3)	C15—H15B	0.9600
La—O4	2.449 (3)	C15—H15C	0.9600
La—O2	2.464 (3)	C16—C17	1.518 (6)
La—N4	2.541 (4)	C16—H16A	0.9600
La—N6	2.577 (4)	C16—H16B	0.9600
La—N5	2.605 (4)	C16—H16C	0.9600
La—O3	2.801 (3)	C17—C22	1.372 (7)
La—O1	2.823 (3)	C17—C18	1.382 (7)
La—O5	2.831 (3)	C18—C19	1.370 (6)
N4—C46	1.109 (7)	C18—H18A	0.9300
N5—C47	1.143 (5)	C19—C20	1.361 (6)
N6—C48	1.134 (5)	C19—H19A	0.9300
N1—C8	1.301 (6)	C20—C21	1.378 (6)
N1—C5	1.408 (6)	C21—C22	1.380 (6)
N1—H1D	0.8600	C21—H21A	0.9300
N2—C23	1.297 (5)	C22—H22A	0.9300
N2—C20	1.405 (5)	C23—C24	1.413 (5)
N2—H2A	0.8600	C23—H23A	0.9300
N3—C38	1.289 (5)	C24—C29	1.405 (5)
N3—C35	1.420 (5)	C24—C25	1.412 (6)
N3—H3B	0.8600	C25—C26	1.352 (6)
S1—C46	1.656 (7)	C25—H25A	0.9300
S2—C47	1.626 (5)	C26—C27	1.394 (6)
S3—C48	1.631 (5)	C26—H26A	0.9300
O1—C13	1.372 (6)	C27—C28	1.369 (6)
O1—C15	1.429 (5)	C27—H27A	0.9300
O2—C14	1.298 (5)	C28—C29	1.415 (6)
O3—C28	1.385 (5)	C30—H30A	0.9600
O3—C30	1.439 (5)	C30—H30B	0.9600
O4—C29	1.299 (5)	C30—H30C	0.9600
O5—C43	1.370 (5)	C31—C32	1.511 (6)
O5—C45	1.434 (5)	C31—H31A	0.9600
O6—C44	1.298 (5)	C31—H31B	0.9600
C1—C2	1.533 (8)	C31—H31C	0.9600
C1—H1A	0.9600	C32—C33	1.355 (6)
C1—H1B	0.9600	C32—C37	1.393 (7)
C1—H1C	0.9600	C33—C34	1.375 (6)
C2—C3	1.386 (8)	C33—H33A	0.9300
C2—C7	1.383 (8)	C34—C35	1.374 (5)
C3—C4	1.353 (7)	C34—H34A	0.9300
C3—H3A	0.9300	C35—C36	1.382 (6)
C4—C5	1.373 (7)	C36—C37	1.381 (6)
C4—H4A	0.9300	C36—H36A	0.9300
C5—C6	1.377 (6)	C37—H37A	0.9300
C6—C7	1.383 (7)	C38—C39	1.406 (6)
C6—H6A	0.9300	C38—H38A	0.9300
C7—H7A	0.9300	C39—C40	1.408 (6)
C8—C9	1.403 (6)	C39—C44	1.420 (6)
C8—H8A	0.9300	C40—C41	1.355 (7)
C9—C14	1.408 (6)	C40—H40A	0.9300
C9—C10	1.434 (7)	C41—C42	1.388 (7)
C10—C11	1.341 (8)	C41—H41A	0.9300
C10—H10A	0.9300	C42—C43	1.348 (6)
C11—C12	1.392 (8)	C42—H42A	0.9300
C11—H11A	0.9300	C43—C44	1.409 (5)
C12—C13	1.358 (7)	C45—H45A	0.9600
C12—H12A	0.9300	C45—H45B	0.9600
C13—C14	1.415 (6)	C45—H45C	0.9600
C15—H15A	0.9600		
			
O6—La—O4	142.38 (9)	O1—C15—H15C	109.5
O6—La—O2	134.32 (10)	H15A—C15—H15C	109.5
O4—La—O2	74.47 (9)	H15B—C15—H15C	109.5
O6—La—N4	73.19 (14)	C17—C16—H16A	109.5
O4—La—N4	110.61 (15)	C17—C16—H16B	109.5
O2—La—N4	127.91 (15)	H16A—C16—H16B	109.5
O6—La—N6	87.25 (12)	C17—C16—H16C	109.5
O4—La—N6	78.93 (12)	H16A—C16—H16C	109.5
O2—La—N6	73.09 (11)	H16B—C16—H16C	109.5
N4—La—N6	158.08 (16)	C22—C17—C18	118.1 (5)
O6—La—N5	73.14 (11)	C22—C17—C16	119.5 (5)
O4—La—N5	70.06 (11)	C18—C17—C16	122.5 (5)
O2—La—N5	138.62 (11)	C19—C18—C17	120.5 (5)
N4—La—N5	85.07 (18)	C19—C18—H18A	119.7
N6—La—N5	79.74 (14)	C17—C18—H18A	119.7
O6—La—O3	142.40 (10)	C20—C19—C18	121.2 (5)
O4—La—O3	59.27 (9)	C20—C19—H19A	119.4
O2—La—O3	70.79 (10)	C18—C19—H19A	119.4
N4—La—O3	69.41 (13)	C19—C20—C21	119.0 (4)
N6—La—O3	130.25 (11)	C19—C20—N2	118.7 (4)
N5—La—O3	106.89 (12)	C21—C20—N2	122.2 (4)
O6—La—O1	100.77 (10)	C22—C21—C20	119.8 (5)
O4—La—O1	116.60 (9)	C22—C21—H21A	120.1
O2—La—O1	58.28 (9)	C20—C21—H21A	120.1
N4—La—O1	75.61 (16)	C21—C22—C17	121.3 (5)
N6—La—O1	118.78 (11)	C21—C22—H22A	119.3
N5—La—O1	160.68 (12)	C17—C22—H22A	119.3
O3—La—O1	66.42 (9)	N2—C23—C24	124.5 (4)
O6—La—O5	57.96 (8)	N2—C23—H23A	117.7
O4—La—O5	144.42 (9)	C24—C23—H23A	117.7
O2—La—O5	76.76 (10)	C29—C24—C23	119.6 (4)
N4—La—O5	103.47 (15)	C29—C24—C25	120.5 (4)
N6—La—O5	72.97 (12)	C23—C24—C25	119.9 (4)
N5—La—O5	124.08 (11)	C26—C25—C24	119.5 (4)
O3—La—O5	128.09 (9)	C26—C25—H25A	120.3
O1—La—O5	62.17 (9)	C24—C25—H25A	120.3
C46—N4—La	143.8 (6)	C25—C26—C27	121.7 (5)
C47—N5—La	170.7 (4)	C25—C26—H26A	119.1
C48—N6—La	157.8 (4)	C27—C26—H26A	119.1
C8—N1—C5	128.8 (4)	C28—C27—C26	119.3 (5)
C8—N1—H1D	115.6	C28—C27—H27A	120.4
C5—N1—H1D	115.6	C26—C27—H27A	120.4
C23—N2—C20	128.4 (4)	C27—C28—O3	125.2 (4)
C23—N2—H2A	115.8	C27—C28—C29	121.5 (4)
C20—N2—H2A	115.8	O3—C28—C29	113.3 (4)
C38—N3—C35	128.5 (4)	O4—C29—C24	122.6 (4)
C38—N3—H3B	115.7	O4—C29—C28	119.9 (4)
C35—N3—H3B	115.7	C24—C29—C28	117.5 (4)
C13—O1—C15	117.8 (4)	O3—C30—H30A	109.5
C13—O1—La	115.6 (3)	O3—C30—H30B	109.5
C15—O1—La	126.3 (3)	H30A—C30—H30B	109.5
C14—O2—La	127.1 (3)	O3—C30—H30C	109.5
C28—O3—C30	116.9 (4)	H30A—C30—H30C	109.5
C28—O3—La	114.4 (2)	H30B—C30—H30C	109.5
C30—O3—La	128.3 (3)	C32—C31—H31A	109.5
C29—O4—La	126.6 (3)	C32—C31—H31B	109.5
C43—O5—C45	118.2 (4)	H31A—C31—H31B	109.5
C43—O5—La	116.4 (2)	C32—C31—H31C	109.5
C45—O5—La	125.4 (3)	H31A—C31—H31C	109.5
C44—O6—La	130.0 (3)	H31B—C31—H31C	109.5
C2—C1—H1A	109.5	C33—C32—C37	118.1 (4)
C2—C1—H1B	109.5	C33—C32—C31	122.2 (5)
H1A—C1—H1B	109.5	C37—C32—C31	119.7 (5)
C2—C1—H1C	109.5	C32—C33—C34	122.4 (5)
H1A—C1—H1C	109.5	C32—C33—H33A	118.8
H1B—C1—H1C	109.5	C34—C33—H33A	118.8
C3—C2—C7	118.2 (5)	C35—C34—C33	119.1 (4)
C3—C2—C1	123.1 (7)	C35—C34—H34A	120.4
C7—C2—C1	118.7 (7)	C33—C34—H34A	120.4
C4—C3—C2	120.9 (6)	C34—C35—C36	120.3 (4)
C4—C3—H3A	119.6	C34—C35—N3	122.4 (4)
C2—C3—H3A	119.6	C36—C35—N3	117.2 (4)
C3—C4—C5	120.7 (6)	C35—C36—C37	119.1 (4)
C3—C4—H4A	119.6	C35—C36—H36A	120.5
C5—C4—H4A	119.6	C37—C36—H36A	120.5
C4—C5—C6	120.1 (5)	C36—C37—C32	121.0 (4)
C4—C5—N1	122.7 (5)	C36—C37—H37A	119.5
C6—C5—N1	117.2 (5)	C32—C37—H37A	119.5
C5—C6—C7	119.0 (5)	N3—C38—C39	123.8 (4)
C5—C6—H6A	120.5	N3—C38—H38A	118.1
C7—C6—H6A	120.5	C39—C38—H38A	118.1
C6—C7—C2	121.1 (6)	C40—C39—C38	120.8 (5)
C6—C7—H7A	119.4	C40—C39—C44	119.4 (4)
C2—C7—H7A	119.4	C38—C39—C44	119.8 (4)
N1—C8—C9	123.1 (4)	C41—C40—C39	120.0 (5)
N1—C8—H8A	118.4	C41—C40—H40A	120.0
C9—C8—H8A	118.4	C39—C40—H40A	120.0
C8—C9—C14	119.9 (4)	C40—C41—C42	120.7 (5)
C8—C9—C10	120.4 (5)	C40—C41—H41A	119.7
C14—C9—C10	119.6 (5)	C42—C41—H41A	119.7
C11—C10—C9	119.7 (6)	C43—C42—C41	121.2 (5)
C11—C10—H10A	120.2	C43—C42—H42A	119.4
C9—C10—H10A	120.2	C41—C42—H42A	119.4
C10—C11—C12	121.4 (6)	C42—C43—O5	127.4 (4)
C10—C11—H11A	119.3	C42—C43—C44	120.5 (5)
C12—C11—H11A	119.3	O5—C43—C44	112.1 (4)
C13—C12—C11	120.3 (6)	O6—C44—C43	119.8 (4)
C13—C12—H12A	119.8	O6—C44—C39	121.9 (4)
C11—C12—H12A	119.8	C43—C44—C39	118.2 (4)
C12—C13—O1	126.1 (5)	O5—C45—H45A	109.5
C12—C13—C14	121.2 (5)	O5—C45—H45B	109.5
O1—C13—C14	112.7 (4)	H45A—C45—H45B	109.5
O2—C14—C9	122.4 (4)	O5—C45—H45C	109.5
O2—C14—C13	119.8 (4)	H45A—C45—H45C	109.5
C9—C14—C13	117.8 (4)	H45B—C45—H45C	109.5
O1—C15—H15A	109.5	N4—C46—S1	176.7 (7)
O1—C15—H15B	109.5	N5—C47—S2	177.9 (5)
H15A—C15—H15B	109.5	N6—C48—S3	179.2 (5)
			
O6—La—N4—C46	36.9 (9)	C5—C6—C7—C2	0.2 (7)
O4—La—N4—C46	177.3 (9)	C3—C2—C7—C6	0.2 (8)
O2—La—N4—C46	−96.7 (9)	C1—C2—C7—C6	−180.0 (5)
N6—La—N4—C46	64.6 (12)	C5—N1—C8—C9	175.3 (4)
N5—La—N4—C46	110.7 (10)	N1—C8—C9—C14	−2.7 (7)
O3—La—N4—C46	−139.1 (10)	N1—C8—C9—C10	−179.9 (5)
O1—La—N4—C46	−69.3 (10)	C8—C9—C10—C11	177.5 (6)
O5—La—N4—C46	−13.2 (10)	C14—C9—C10—C11	0.2 (9)
O6—La—N6—C48	−94.2 (11)	C9—C10—C11—C12	−0.8 (11)
O4—La—N6—C48	121.0 (11)	C10—C11—C12—C13	0.1 (11)
O2—La—N6—C48	44.0 (10)	C11—C12—C13—O1	−176.9 (6)
N4—La—N6—C48	−120.7 (11)	C11—C12—C13—C14	1.1 (9)
N5—La—N6—C48	−167.6 (11)	C15—O1—C13—C12	−10.9 (7)
O3—La—N6—C48	88.9 (11)	La—O1—C13—C12	163.0 (4)
O1—La—N6—C48	6.6 (11)	C15—O1—C13—C14	170.9 (4)
O5—La—N6—C48	−37.0 (10)	La—O1—C13—C14	−15.1 (5)
O6—La—O1—C13	154.7 (3)	La—O2—C14—C9	−153.9 (3)
O4—La—O1—C13	−29.8 (3)	La—O2—C14—C13	26.3 (6)
O2—La—O1—C13	18.8 (3)	C8—C9—C14—O2	3.8 (7)
N4—La—O1—C13	−135.9 (3)	C10—C9—C14—O2	−178.9 (5)
N6—La—O1—C13	62.0 (3)	C8—C9—C14—C13	−176.4 (4)
N5—La—O1—C13	−135.7 (4)	C10—C9—C14—C13	0.9 (7)
O3—La—O1—C13	−62.4 (3)	C12—C13—C14—O2	178.2 (5)
O5—La—O1—C13	110.1 (3)	O1—C13—C14—O2	−3.5 (6)
O6—La—O1—C15	−31.9 (4)	C12—C13—C14—C9	−1.6 (7)
O4—La—O1—C15	143.6 (4)	O1—C13—C14—C9	176.6 (4)
O2—La—O1—C15	−167.8 (4)	C22—C17—C18—C19	−1.1 (8)
N4—La—O1—C15	37.4 (4)	C16—C17—C18—C19	179.3 (5)
N6—La—O1—C15	−124.7 (4)	C17—C18—C19—C20	0.5 (8)
N5—La—O1—C15	37.6 (6)	C18—C19—C20—C21	0.4 (7)
O3—La—O1—C15	110.9 (4)	C18—C19—C20—N2	−177.3 (4)
O5—La—O1—C15	−76.6 (4)	C23—N2—C20—C19	165.2 (4)
O6—La—O2—C14	−96.2 (3)	C23—N2—C20—C21	−12.4 (7)
O4—La—O2—C14	112.5 (3)	C19—C20—C21—C22	−0.7 (7)
N4—La—O2—C14	8.2 (4)	N2—C20—C21—C22	176.9 (4)
N6—La—O2—C14	−164.6 (4)	C20—C21—C22—C17	0.1 (7)
N5—La—O2—C14	144.2 (3)	C18—C17—C22—C21	0.8 (8)
O3—La—O2—C14	50.2 (3)	C16—C17—C22—C21	−179.6 (5)
O1—La—O2—C14	−23.4 (3)	C20—N2—C23—C24	−177.0 (4)
O5—La—O2—C14	−88.7 (3)	N2—C23—C24—C29	1.1 (6)
O6—La—O3—C28	−157.1 (3)	N2—C23—C24—C25	−179.5 (4)
O4—La—O3—C28	−19.5 (3)	C29—C24—C25—C26	−0.2 (7)
O2—La—O3—C28	63.4 (3)	C23—C24—C25—C26	−179.6 (4)
N4—La—O3—C28	−150.9 (3)	C24—C25—C26—C27	−1.1 (8)
N6—La—O3—C28	17.7 (3)	C25—C26—C27—C28	0.4 (8)
N5—La—O3—C28	−73.0 (3)	C26—C27—C28—O3	179.4 (4)
O1—La—O3—C28	126.3 (3)	C26—C27—C28—C29	1.4 (7)
O5—La—O3—C28	117.9 (3)	C30—O3—C28—C27	12.8 (7)
O6—La—O3—C30	29.9 (5)	La—O3—C28—C27	−161.1 (4)
O4—La—O3—C30	167.5 (4)	C30—O3—C28—C29	−169.1 (4)
O2—La—O3—C30	−109.6 (4)	La—O3—C28—C29	17.0 (4)
N4—La—O3—C30	36.1 (4)	La—O4—C29—C24	157.7 (3)
N6—La—O3—C30	−155.2 (4)	La—O4—C29—C28	−24.1 (5)
N5—La—O3—C30	114.0 (4)	C23—C24—C29—O4	−0.4 (6)
O1—La—O3—C30	−46.7 (4)	C25—C24—C29—O4	−179.8 (4)
O5—La—O3—C30	−55.1 (4)	C23—C24—C29—C28	−178.7 (4)
O6—La—O4—C29	160.4 (3)	C25—C24—C29—C28	1.9 (6)
O2—La—O4—C29	−53.8 (3)	C27—C28—C29—O4	179.1 (4)
N4—La—O4—C29	71.5 (3)	O3—C28—C29—O4	1.0 (6)
N6—La—O4—C29	−129.1 (3)	C27—C28—C29—C24	−2.6 (6)
N5—La—O4—C29	147.9 (3)	O3—C28—C29—C24	179.2 (4)
O3—La—O4—C29	22.8 (3)	C37—C32—C33—C34	0.9 (7)
O1—La—O4—C29	−12.3 (3)	C31—C32—C33—C34	−179.0 (4)
O5—La—O4—C29	−90.9 (3)	C32—C33—C34—C35	0.0 (7)
O6—La—O5—C43	−15.0 (3)	C33—C34—C35—C36	−0.8 (6)
O4—La—O5—C43	−152.0 (3)	C33—C34—C35—N3	−179.9 (4)
O2—La—O5—C43	171.3 (3)	C38—N3—C35—C34	−8.2 (7)
N4—La—O5—C43	44.9 (3)	C38—N3—C35—C36	172.7 (4)
N6—La—O5—C43	−112.6 (3)	C34—C35—C36—C37	0.8 (6)
N5—La—O5—C43	−48.2 (3)	N3—C35—C36—C37	179.9 (4)
O3—La—O5—C43	119.2 (3)	C35—C36—C37—C32	0.0 (7)
O1—La—O5—C43	110.4 (3)	C33—C32—C37—C36	−0.9 (7)
O6—La—O5—C45	168.4 (4)	C31—C32—C37—C36	179.0 (4)
O4—La—O5—C45	31.5 (4)	C35—N3—C38—C39	−179.2 (4)
O2—La—O5—C45	−5.3 (4)	N3—C38—C39—C40	176.7 (5)
N4—La—O5—C45	−131.6 (4)	N3—C38—C39—C44	−1.4 (7)
N6—La—O5—C45	70.8 (4)	C38—C39—C40—C41	−178.2 (6)
N5—La—O5—C45	135.2 (4)	C44—C39—C40—C41	−0.1 (8)
O3—La—O5—C45	−57.4 (4)	C39—C40—C41—C42	0.3 (10)
O1—La—O5—C45	−66.2 (4)	C40—C41—C42—C43	−0.7 (10)
O4—La—O6—C44	156.8 (3)	C41—C42—C43—O5	−179.2 (5)
O2—La—O6—C44	25.9 (4)	C41—C42—C43—C44	0.9 (8)
N4—La—O6—C44	−101.1 (4)	C45—O5—C43—C42	10.2 (7)
N6—La—O6—C44	88.9 (4)	La—O5—C43—C42	−166.6 (4)
N5—La—O6—C44	169.1 (4)	C45—O5—C43—C44	−169.8 (4)
O3—La—O6—C44	−95.0 (4)	La—O5—C43—C44	13.4 (5)
O1—La—O6—C44	−29.8 (4)	La—O6—C44—C43	−18.0 (6)
O5—La—O6—C44	17.4 (3)	La—O6—C44—C39	162.5 (3)
C7—C2—C3—C4	−0.2 (9)	C42—C43—C44—O6	179.8 (5)
C1—C2—C3—C4	179.9 (6)	O5—C43—C44—O6	−0.1 (6)
C2—C3—C4—C5	−0.1 (9)	C42—C43—C44—C39	−0.7 (7)
C3—C4—C5—C6	0.4 (8)	O5—C43—C44—C39	179.4 (4)
C3—C4—C5—N1	−177.7 (5)	C40—C39—C44—O6	179.8 (4)
C8—N1—C5—C4	−20.3 (7)	C38—C39—C44—O6	−2.1 (7)
C8—N1—C5—C6	161.5 (5)	C40—C39—C44—C43	0.3 (7)
C4—C5—C6—C7	−0.5 (7)	C38—C39—C44—C43	178.4 (4)
N1—C5—C6—C7	177.7 (4)		

### Hydrogen-bond geometry (Å, °)

**Table:**

*D*—H···*A*	*D*—H	H···*A*	*D*···*A*	*D*—H···*A*
N1—H1D···O2	0.86	1.86	2.559 (4)	138
N2—H2A···O4	0.86	1.90	2.588 (4)	136
N3—H3B···O6	0.86	1.87	2.570 (4)	138
